# Sexual Dimorphism of Corticosteroid Signaling during Kidney Development

**DOI:** 10.3390/ijms22105275

**Published:** 2021-05-18

**Authors:** Margaux Laulhé, Laurence Dumeige, Thi An Vu, Imene Hani, Eric Pussard, Marc Lombès, Say Viengchareun, Laetitia Martinerie

**Affiliations:** 1 Université Paris-Saclay, Inserm, Physiologie et Physiopathologie Endocriniennes, CEDEX, 94276 Le Kremlin-Bicêtre, France; margaux.laulhe@universite-paris-saclay.fr (M.L.); laurence.dumeige@aphp.fr (L.D.); thi-an.vu@universite-paris-saclay.fr (T.A.V.); imene.hani@universite-paris-saclay.fr (I.H.); eric.pussard@aphp.fr (E.P.); marc.lombes@universite-paris-saclay.fr (M.L.); say.viengchareun@universite-paris-saclay.fr (S.V.); 2 Pediatric Endocrinology Department, Hôpital Universitaire Robert Debre, France & Université de Paris, 75019 Paris, France; 3 Service de Génétique Moléculaire, Pharmacogénétique et Hormonologie, Hôpital de Bicêtre, Assistance Publique-Hôpitaux de Paris, 94275 Le Kremlin-Bicêtre, France

**Keywords:** aldosterone, cortisol, mineralocorticoid and glucocorticoid receptors, neonates, kidney, development, sexual dimorphism

## Abstract

Sexual dimorphism involves differences between biological sexes that go beyond sexual characteristics. In mammals, differences between sexes have been demonstrated regarding various biological processes, including blood pressure and predisposition to develop hypertension early in adulthood, which may rely on early events during development and in the neonatal period. Recent studies suggest that corticosteroid signaling pathways (comprising glucocorticoid and mineralocorticoid signaling pathways) have distinct tissue-specific expression and regulation during this specific temporal window in a sex-dependent manner, most notably in the kidney. This review outlines the evidence for a gender differential expression and activation of renal corticosteroid signaling pathways in the mammalian fetus and neonate, from mouse to human, that may favor mineralocorticoid signaling in females and glucocorticoid signaling in males. Determining the effects of such differences may shed light on short term and long term pathophysiological consequences, markedly for males.

## 1. Introduction

Corticosteroids (mineralocorticosteroids and glucocorticosteroids) are crucial hormones implicated in the function of many tissues to maintain homeostasis. Their major actions rely on their binding to the Mineralocorticoid and Glucocorticoid receptors (MR and GR, respectively). Recent studies have emphasized a particular temporal window during kidney development, which is well-conserved between mammals, where corticosteroid signaling pathways have a specific pattern of expression and regulation, in relation to the adaptation of the fetus and the neonate, transitioning from a water to an air environment. This review will first present a brief description of mineralocorticoid and glucocorticoid signaling pathways (from aldosterone and cortisol biosynthesis to mechanisms of regulation and action of MR and GR) during kidney development. Particular emphasis will be placed on recent studies highlighting a sexually dimorphic expression, which may have a pathophysiological impact, especially in males/boys, who experience increased difficulties to adapt during the neonatal period and are at higher risk of developing early hypertension later in life.

## 2. Mineralocorticoid Signaling Pathway

### 2.1. Regulation of Aldosterone Synthesis

Aldosterone, a steroid hormone secreted by the *Zona Glomerulosa* (*ZG*), the outer layer of the adrenal gland cortex, is vital for maintaining body fluid and electrolyte homeostasis, through sodium retention and thereby controls blood pressure [1]. As the adrenal *ZG* has no capacity to store aldosterone once it is produced, the regulation of its secretion is inextricably linked to transcriptional activation as well as post-transcriptional modifications of steroidogenic enzymes. Acute aldosterone production is controlled by the early regulatory step of cholesterol uptake and conversion to pregnenolone that is mediated by the increased expression and phosphorylation of the steroidogenic acute regulatory protein, StAR (encoded by the *STAR* gene). A late regulatory step controlling the expression of biosynthesis enzymes, particularly CYP11B2 (aldosterone synthase, encoded by *CYP11B2* gene), regulates chronic aldosterone production [2]. Aldosterone biosynthesis in the *ZG* is physiologically regulated by Angiotensin II (Ang II), potassium (K^+^), and at a lesser extent by the AdrenoCorticoTropic Hormone (ACTH). Other bioactive compounds (serotonin, leptin, endothelin, nitric oxide, catecholamines, atrial natriuretic peptide, neuropeptide substance P) released by adipocytes, mast cells, chromaffin cells, or nerve ending located close to *ZG* cells were also shown to stimulate aldosterone secretion [3,4]. Stimulation of the Renin–Angiotensin System (RAS) is initiated by increased sympathetic activity, reduced perfusion pressure in the renal afferent arterioles, or decreased sodium content in the *macula densa* of the renal distal tubules, which lead to the release of renin from juxtaglomerular cells. Afterwards, renin converts circulating angiotensinogen produced by the liver to angiotensin I (Ang I), which is subsequently cleaved by Angiotensin-Converting Enzyme (ACE) to form the octapeptide Ang II. The binding of Ang II to its AT1 receptor (AT1R) triggers the release of calcium from intracellular stores, which is the major determinant of aldosterone secretion [3]. Small increases in extracellular K^+^ depolarize the glomerulosa cell, also increasing calcium influx through voltage-gated calcium channels that stimulates *CYP11B2* and *StAR* transcription [5]. Finally, ACTH alone stimulates aldosterone secretion acutely and transiently but to a lesser extent than Ang II and K^+^. ACTH binding to its Melanocortin Receptor 2 (MC2R) stimulates *StAR* expression through adenylate cyclase activation [6]. During development, fetal aldosterone production occurs in the definitive zone, which is the counterpart of the *ZG* of the adult adrenal cortex. While *StAR* and other important enzymes expression gradually rise during pregnancy [7], *CYP11B2* expression only appears around 24 gestational weeks (GW) [8]; then, it increases to reach at birth similar levels than in the adult adrenals [9]. Detectable plasma concentrations of aldosterone are found in premature newborns as early as 25 GW [10], but aldosterone production remains low until 30 GW [9]. Aldosterone concentration increases thereafter until term [10], in relation to a fetal neo-synthesis [11]. No sexual dimorphism has been demonstrated concerning plasma aldosterone levels in the fetus or at birth [12].

### 2.2. The Mineralocorticoid Receptor (MR)

#### 2.2.1. Gene, Transcripts, and Protein Variants

MR belongs to the nuclear receptor superfamily that mediates sodium-retaining action of aldosterone in the distal nephron [13]. This transcription factor is encoded by the *NR3C2* gene, which is located in humans at locus 4q31.1–4q31.2 [14,15] and encodes a 984 amino-acids protein (≈107 kDa) [16], organized into four distinct structural domains: the N-terminal domain (NTD), the DNA binding domain (DBD), the hinge region, and the ligand binding domain (LBD). MR functions were shown to be modulated by splice variants, lacking either exon 6 or both exon 5 and 6 [17,18]. Two major variants of human MR, named MRA and MRB, are generated by alternative initiation sites of translation from methionine 1 and 15, respectively. These MR variants display distinct transactivation capacities in vitro [19].

#### 2.2.2. Mechanisms of Regulation of MR Expression and Activity

Two alternative promoters drive expression of the *NR3C2* gene [20], the proximal P1 promoter, which is transcriptionally active in all MR target tissues, and the distal P2 promoter, which is weaker and transcriptionally active in the central nervous system during specific developmental stages or physiological situations [21]. Of particular interest, the expression of this nuclear receptor, which transcriptionally regulates water and sodium balance, is also controlled at the post-transcriptional level by osmotic tone, notably in the distal parts of the nephron, where large variations of extracellular tonicity prevail [22]. Indeed, MR transcript levels decrease under hypertonicity following recruitment of the RNA Binding Protein (RBP) Tis11b (tetradecanoyl phorbol acetate inducible sequence 11b), which physically interacts with 3′-untranslated region (3′-UTR) of MR transcript, thus modulating its mRNA turnover in response to osmotic stress [23]. On the opposite, MR transcript levels increase under hypotonicity thanks to the recruitment of Human antigen R (HuR), another RBP, which interacts with MR 3′-UTR in the cytoplasm of renal cells to stabilize and increase MR levels, thereby modulating MR signaling [24]. Accumulating evidence now underscores the pivotal role of microRNAs (miRNAs), an additional class of post-transcriptional regulators, in the control of MR expression in the kidney [25,26]. Beyond these regulatory mechanisms, MR activity and signaling are also modulated by post-translational modifications such as ubiquitylation, SUMOylation, phosphorylation, and acetylation [13,27].

## 3. Glucocorticoid Signaling Pathway

### 3.1. Glucocorticoid Hormones and the Hypothalamic–Pituitary–Adrenal Axis

Glucocorticoid hormones (cortisol and corticosterone in rodents) are the effector hormones of the Hypothalamic–Pituitary–Adrenal (HPA) axis of the neuroendocrine system and are produced by the adrenal *Zona Fasciculata* (*ZF*). As for all steroid hormones, cortisol synthesis begins from cholesterol and is critically dependent on the StAR protein, which facilitates a rapid flux of cholesterol into mitochondria. Then, the mitochondrial enzyme, cytochrome P450scc, encoded by the *CYP11A1* gene cleaves cholesterol sidechain to pregnenolone. Pregnenolone passively diffuses into the endoplasmic reticulum and is converted to progesterone by the 2–3β-hydroxysteroid dehydrogenase/Δ5-Δ4 isomerase (3βHSD2), which is encoded by the *HSD3B2* gene. The specific expression of P450c17 (encoded by *CYP17A1* gene) catalyzes the 17α-hydroxylation of progesterone to 17OH-progesterone (17OHP). Thereafter, 17OHP is successively converted to 11-deoxycortisol then to cortisol by the microsomal P450c21 and mitochondrial P450c11β (encoded by *CYP11B1* gene), respectively. In rodents, the *ZF* lacks P450c17 and progesterone is 21- and 11β-hydroxylated to yield corticosterone, instead of cortisol, as the dominant glucocorticoid in these species [2]. Glucocorticoid synthesis is differentially regulated in the pre- and postnatal adrenal glands. In the adult, glucocorticoid production is critically controlled by the activity of the HPA axis. Various stimuli such as stress, illness, or the circadian rhythm activates the release of Corticotropin-Releasing Hormone (CRH) from the hypothalamus, which stimulates the anterior pituitary gland, releasing ACTH. ACTH acts on MC2R in the adrenal *ZF* to induce corticosteroid synthesis from cholesterol. In turn, circulating glucocorticoids exert a feedback regulatory effect on the hypothalamus and on the pituitary to inhibit the release of CRH and ACTH, respectively [2]. Fetal adrenal glands are capable of steroidogenesis soon after their formation around the 7th GW. At the same time, the pituitary begins to produce ACTH. The secretion of cortisol increases to a peak at 8–9 GW; then, it decreases until 14 GW. This cyclic secretion of glucocorticoids by the fetal adrenal glands is not under control of the ACTH, as in adults [28]. Indeed, ACTH levels remain constant during this period and stimulate the adrenal glands to produce androgens. The expression of 3βHSD2 that peaks at 9 GW decreases thereafter throughout most of the second trimester, leading to a reduction in glucocorticoid synthesis. At 24 GW, the expression of 3βHSD2 and secretion of glucocorticoids resume. Cortisol surges in the weeks prior to birth and plays crucial roles in the differentiation and functional development of several organs such as the lungs [29]. Sexual dimorphism in HPA axis activity has been suggested to be present in early childhood. In a meta-analysis, basal HPA axis activity was suggested to be greater among boys before 8 years of age, as assessed by salivary cortisol levels [30]. After 8 years, this trend seemed to reverse, suggesting a sex-specific evolution of cortisol metabolism around puberty [31] and a possible effect of early life programming [32]. However, no difference was observed at birth between girls and boys regarding basal plasma cortisol levels [33]. Cortisol metabolism relies on the activity of liver A-ring reductases (5-α and 5-β-reductase) and 11beta-hydroxysteroid dehydrogenase (11βHSD) isoenzymes. The 11βHSD1 enzyme is mainly expressed in the liver and adipose tissue, and it regenerates cortisol from its inactive compound cortisone. The 11βHSD2 enzyme catalyzes the reverse reaction in renal epithelial cells (see Section 4.2). In adulthood, females were found to have a lower urinary excretion rate of cortisol metabolites in comparison to males, which was attributed to a less A-ring reduction [34]. This sex difference in cortisol metabolism begins around puberty, at the age of 10–11 years [31,35], and it is maintained in elderly subjects suggesting regulatory mechanisms partially independent from gonadal steroids [36].

### 3.2. The Glucocorticoid Receptor

#### 3.2.1. Gene, Transcripts, and Protein Variants

GR is the founding member of the nuclear receptor superfamily. This transcription factor, similar to the MR, contains 4 main domains, the NTD, DBD, LBD and a Hinge Region (HR) between the DBD and the LBD [37]. It is encoded by the *NR3C1* gene located on chromosome 5 (5q31) in humans. The *NR3C1* gene contains at least 10 exons [38]. The alternative splicing of exon 9 produces the two major variants of the protein, GRα, which is the active ligand-dependent variant and GRβ, which is a ligand-independent variant exerting dominant negative effect [39]. The GRα is a 777 amino acids length protein [40]. The GR protein contains sites for post-translational modifications, such as SUMOylation or phosphorylation, which influence its transactivation capacities.

#### 3.2.2. Mechanisms of Regulation of GR Expression and Activity

GR is present in virtually all the cells, but sensitivity to glucocorticoids is tissue-dependent and partially mediated by regulation of GR expression. This regulation is mediated at the transcriptional level by two main mechanisms: alternative splicing of the 1st exon and variability in the length of the N-terminal domain. The first and untranslated exon contains nine splice donor sites well-preserved between species, corresponding to splice recipient sites on the exon 2 [41] each under the control of a specific promoter. This variability results in alternative mRNA isoforms, which differs in their 5′-UTR regions. The expression of those mRNA isoforms is tissue specific. The 2nd exon contains eight different start codons, encoding for eight variants of the GR (GR-A, GR-B, GR-C1, GR-C2, GR-C3, GR-D1, GR-D2, and GR-D3). These variants have equal affinity for the ligand but differ in transactivation capacities and target genes, with only <10% of them common to all variants [42].

## 4. Mechanism of Corticosteroids Action in Renal Principal Cells

### 4.1. Subcellular Distribution

In renal principal cells, corticosteroid hormones enter by passive diffusion and bind their respective receptor: aldosterone to the MR and cortisol (or corticosterone) to the GR. In the absence of ligands, corticosteroid receptors are associated to chaperone proteins [43,44,45,46], which protect receptors from degradation and maintain a conformation suitable for binding to ligands. Thereafter, the binding of either ligand induces the dissociation of these chaperone proteins and conformational changes of MR and GR.

### 4.2. Mineralocorticoid Selectivity

**Figure 1:**
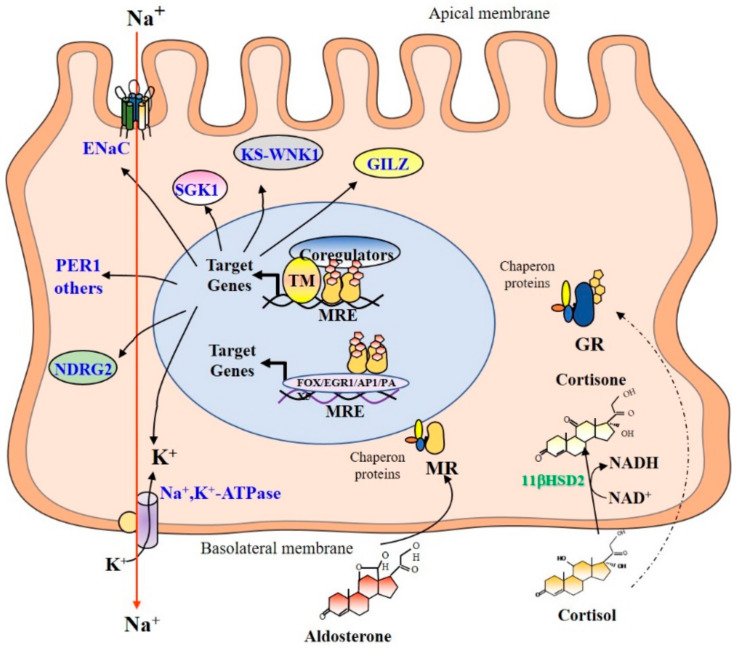
Mineralocorticoid and glucocorticoid signaling in renal principal cells. Corticosteroid hormones enter by passive diffusion and bind their respective receptor: aldosterone to MR and cortisol/corticosterone to GR. In the absence of ligands, corticosteroid receptors are associated to chaperone proteins. Thereafter, the binding of either ligand induces the dissociation of these chaperone proteins and conformational changes of MR and GR. In the nucleus, the aldosterone/MR complex binds mostly as homodimers to Mineralocorticoid Response Elements (MREs). Then, MR interacts, in a cyclic, sequential, and/or combinatorial manner, with transcriptional coregulators and some basal transcription factors or components of the machinery to enhance the transcription of target genes, including the Epithelial Na^+^ Channel (ENaC), the Na^+^, K^+^-ATPase pump. Aldosterone also stimulates early expression of the *Serum and glucocorticoid-regulated kinase 1* (*SGK1*), the *serine/threonine kinase With No lysine K kinase* (*KS-WNK1*), the *N-myc Down-Regulated Gene 2* (*NDRG2*), and the *Glucocorticoid-Induced Leucine Zipper protein* (*GILZ*). Recently, aldosterone was shown to stimulate early expression the *PER1* gene, which belongs to the circadian clock gene family. It was also reported that MR can indirectly bind to recognition motifs for other transcription factors (FOX, EGR1, AP1, PAX5) through tethering mechanisms. In principal renal cells, the 11βHSD2 converts glucocorticoid hormones into cortisone or 11-dehydrocorticosterone that have little or no affinity for MR, or even for GR. Thus, the 11βHSD2 allows aldosterone to act selectively onto MR to specifically exert its biological effects on sodium reabsorption. In addition, GR is not or weakly activated. MR: Mineralocorticoid Receptor; GR: Glucocorticoid Receptor; MRE: Mineralocorticoid Response Element; GILZ: Glucocorticoid-induced leucine zipper; ENaC: Epithelial Na^+^ Channel; Sgk 1: Serum and Glucocorticoid-Regulated kinase 1; KS-WNK1: With No lysine K kinase; NDRG2: N-myc Down-Regulated Gene 2; PER 1: clock gene period 1; TM: Transcriptional Machinery.

Given the homology existing between the structure of aldosterone and cortisol/corticosterone, the high homology between MR and GR (their DBD and LBD have 94% and 57% homology, respectively [47]), and similar affinity of both receptors for glucocorticoid hormones, MR would be expected to be permanently occupied by glucocorticoid hormones. Indeed, cortisol plasma concentrations are up to 100 to 1000 times higher than that of aldosterone in mammals. However, MR illicit occupation by glucocorticoid hormones is limited in renal principal and other epithelial cells by the action of the 11βHSD2 [48,49,50]. This enzyme oxidizes the alcohol function carried by carbon 11 of the glucocorticoid hormones into a ketone function, thus producing 11βdehydrogenated derivatives (cortisone in humans and 11-dehydrocorticosterone in rodents) that have little or no affinity for MR, or even for GR [48]. Thus, the 11βHSD2 allows aldosterone to act selectively onto MR in epithelial cells to specifically exert its biological effects on sodium reabsorption (Figure 1). In addition, MR can also discriminate between aldosterone and cortisol because dissociation rates are much faster for glucocorticoids than for aldosterone. Distinct interactions between the NTD and the LBD occur because the aldosterone–MR complex adopts structural conformation somehow different from that of the glucocorticoid–MR complex [51]. Finally, it has been shown that the nature of the ligand might also modify the cyclicality of the interaction between ligand–receptor complex with DNA responsive elements [52].

### 4.3. Promoter Binding and Recruitment of Coregulators 

Once in the nucleus, the aldosterone–MR complex binds mostly as homodimers to Mineralocorticoid Response Elements (MREs) located in the regulatory regions of MR target genes [53]. Then, MR interacts, in a cyclic, sequential, and/or combinatorial manner [52], with transcriptional coregulators [54] and some basal transcription factors or components of the machinery to enhance transcriptional activation and to facilitate chromatin remodeling involving histone acetylation/methylation [53]. Interestingly, Le Billan et al., used HK-GFP-MR cells, a human renal cell line that is devoid of 11βHSD2 to decipher the respective contribution of MR/GR and aldosterone/cortisol in renal corticosteroid signaling. These authors provided evidence that MR and GR dynamically and cyclically interact at the same target promoter on the *Period circadian protein 1* (*PER1*) gene, in a specific and distinct transcriptional signature, by binding as homo- or heterodimers [52]. In the nucleus, the GR can also bind specific sequences called Glucocorticoid Response Elements (GREs). In each cell type, GR binds different GREs. Binding to the GRE activates the recruitment of chromatin-remodeling complexes and coregulators, such as steroid receptor coactivator-1 (SRC-1), which allows the formation of the transcription initiation complex. Negative GRE (nGRE) were also reported, which are responsible for trans-repression of the target genes. Binding to nGRE prevents the dimerization and allows for the recruitment of corepressors, such as NCoR or SMRT [55]. GR can also mediate trans-repression of target genes by tethering as described for the MR [53] via interactions with other transcription factors as NF-κB or AP-1 without direct binding to DNA [56].

### 4.4. MR and GR Target Genes

In the aldosterone-sensitive nephron, MR participates in the control of salt balance by stimulating expression of ionic transporters such as the Epithelial Na^+^ Channel (ENaC) [57] and the Na^+^, K^+^-ATPase pump [58]. These transporters enable the transepithelial reabsorption of sodium from the lumen to the *interstitium*. Aldosterone also stimulates, via MR activation, early expression of the Serum and glucocorticoid-regulated kinase 1 (SGK1) [59], which phosphorylates the ubiquitin ligase Nedd4-2, which in turn controls the retrieval of ENaC from the apical membrane. Other target genes have also been identified in the kidney, including the *serine/threonine kinase With No lysine K kinase* (KS-WNK1) [60], the *N-myc Down-Regulated Gene 2* (*NDRG2*) [61], the *Glucocorticoid-Induced Leucine Zipper protein* (*GILZ*) [62], which also play pivotal roles during the early phase of aldosterone responses [13] (Figure 1). Recently, aldosterone was shown to regulate the rhythmicity of renal sodium reabsorption by stimulating the early expression of the *PER1* gene [63]. It was also reported that MR can indirectly bind to recognition motifs for other transcription factors (FOX, EGR1, AP1, PAX5) through tethering mechanisms, as reported for the GR, thus enabling the modulation of target gene expression [53]. In adult kidney, as 11βHSD2 expression is high, no major effect of GR signaling is expected under basal conditions [64]. Importantly, our group has recently identified a specific temporal window during renal development, during which this MR signaling is ineffective due to the down-regulation of MR expression [65]. Therefore, given that renal 11βHSD2 is not expressed during this specific perinatal period, GR signaling should be functional in renal principal cells, with plasma cortisol levels detectable in physiological amounts in newborns similar to adult levels [66]. In this context, GR is likely to activate specific renal target genes as well as common target genes with those of the MR including *SGK1* or *GILZ*. GR and MR-specific target genes in renal principal cells are summarized in Table 1.

## 5. Sexual Dimorphism of Corticosteroid Signaling Aside from the Kidney

Several studies have provided evidence for a gender differential expression and activation of MR and GR. For instance, repeated antenatal glucocorticoid treatment was shown to program HPA function in a sex-specific manner, and these changes were associated with the modification of MR and GR expression in the adult brain and pituitary [68]. During development, the same authors observed decreased GR mRNA in the paraventricular nucleus, decreased MR mRNA and MR protein in the hippocampus, and increased GR mRNA and GR protein in the hippocampus. In guinea pig, maternally administered glucocorticoids reduced fetal plasma ACTH and cortisol concentrations and significantly affected hippocampal MR protein expression, and this effect was greatest in males. The sex differences in the pattern of GR and MR expression during development may indicate different windows of vulnerability to prenatal glucocorticoid exposure in fetal life [69]. These corticosteroid receptors were also shown to play a pivotal role in the modulation of stress response in the rat brain. Indeed, the contribution of gender and of the cellular environment of certain brain areas to the expression of MR and GR was reported following restraint stress [70]. Furthermore, the same group observed that female rats presented with a distinct mechanism of regulating GR/MR ratio in the hippocampus upon chronic stress, while the female hypothalamus was more prone than the male to changing corticosteroid receptor expression in response to restraint stress. Few other studies have also reported gender differences in MR expression and activation in the heart [71,72,73]. Similarly, it has been shown that glucocorticoids exert their actions, notably anti-inflammatory activity, in a sexually dimorphic manner [74,75]. In addition, estrogens can antagonize GR-induction of the *GILZ* gene [76]. Whether the regulatory mechanisms implicated in MR and GR expression, or their coregulators expression, could contribute to the emergence of a sexual dimorphism remain to be explored. Finally, to the best our knowledge, only one study has reported a sexual dimorphism for corticosteroid receptors expression in the kidney [77].

## 6. Gender Differences in Kidney Development and Organogenesis

Kidney organogenesis is a complex process involving three successive structures, of which only the last one, the metanephros, will give the definitive kidney [78]. The metanephros develops from the caudal nephrotomes starting from the 5th GW, and its maturation continues until the end of the first year of postnatal life in humans [65], with parallel maturation of the nephrons and of the different parts of the collecting ducts [79]. Renal ontogeny starts by the interaction between the mesenchymal cells of the metanephros, which will give the future nephronic structures, and the ureteral bud, an epithelial structure developed from the Wolffian duct, from which the renal collecting system will develop by successive dichotomies, according to a classical branching morphogenesis [80]. Each branch from the ureteral bud is capped by metanephronic cells, which are progenitor stem cells that are capable of differentiating into all the cell types composing the glomeruli and nephrons [81]. These differentiation mechanisms are possible thanks to a dialogue between the two structures and the successive expression of different signaling pathways [82,83], some of which are epigenetically regulated [84] and thus potentially impacted by adverse events occurring during pregnancy. In particular, Ang II, acting on the AT1R, mediates the growth and proliferation of renal tubules and branching morphogenesis [85]. In contrast, the AT2 Receptor (AT2R) in the fetal kidney has anti-proliferative actions in the renomedullary interstitial cells and acts to mediate apoptosis [86]. All these processes are of crucial importance in determining the final number of nephrons per kidney, which is directly correlated with renal function in adulthood. Nephrogenesis is essentially antenatal [87], between the 5th and 36th GW, but more particularly between the 17th and 32nd GW, resulting in a total number of nephrons in humans between 300,000 and 1.1 million [88]. From studies carried out on autopsies or donor kidneys, it is known that there is a sexual dimorphism in renal measurements in adulthood, both in absolute values and in relative values in relation to the body surface area, with significantly higher values in men [89,90]. This implies that the number of total nephrons could be higher in males than in females, although this has not been formally proven in the human species. Interestingly, this sexual dimorphism arises early during nephrogenesis, since differences in renal volume have been found in ultrasound measurements in the fetus during the third trimester of pregnancy, as well as in infants up to 4 years of age [91,92,93]. On the other hand, no sexual dimorphism was found in terms of nephron counts in the neonatal period [94], but these data have been scarcely studied, on very small cohorts. Renal ontogeny in the mouse is relatively similar to that of the human species, with the succession of three structures, pronephros from the 8th day of gestation (E8), mesonephros from E9, and the metanephros from E11. The main difference is that in the murine species, nephrogenesis continues postnatally until the end of the first week of life. Moreover, sexual dimorphism in renal volume does not exist in the neonatal period in mice [95], which is possibly related to this delay in the acquisition of new nephrons. However, it appears significantly, concomitantly with histological structural changes, starting from the 50th day of life, i.e., after the onset of puberty in mice [95]. A direct effect of testosterone on renal volume has been demonstrated in mouse models of young castrated males secondarily exposed to testosterone or vehicle [96]. The trophic effect of testosterone on organ development, including the kidney, has also been demonstrated in human clinical studies [97]. Thus, sexual dimorphism in renal organogenesis as early as the third trimester in the human species could be related to testosterone secretion by male fetuses in utero [98]. Studies of prenatal exposure to testosterone have shown that the developing kidney is sensitive to testosterone [99], but its implication on renal development under physiological conditions in the fetus, as well as the molecular interaction between androgen signaling pathway and other signaling pathways implicated in nephrogenesis, remain to be further demonstrated.

## 7. Particularities of Mineralocorticoid and Glucocorticoid Signalings during Renal Development

**Figure 2:**
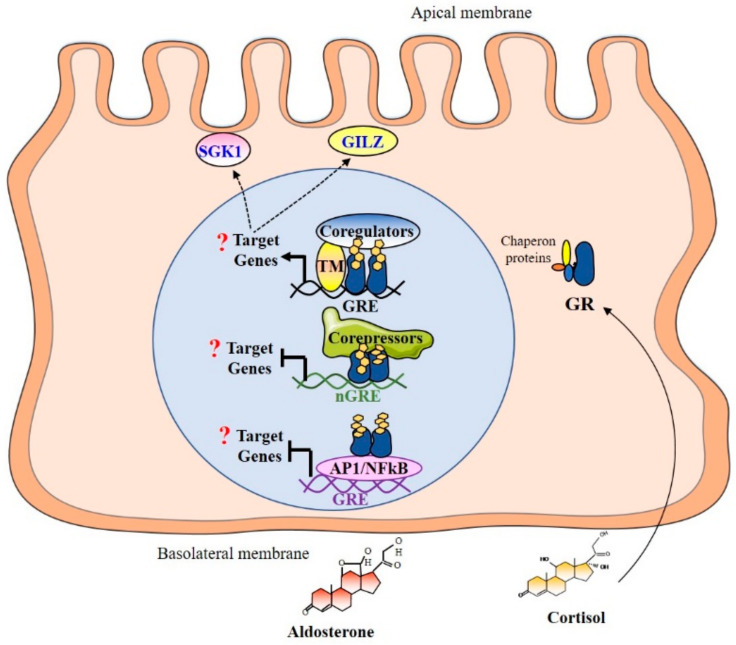
Mineralocorticoid and glucocorticoid signaling in renal principal cell during the perinatal period. MR signaling pathway is ineffective during renal development due to the down-regulation of MR expression. Given that renal 11βHSD2 is not expressed during this specific perinatal period, the GR signaling pathway is therefore functional. In the nucleus, the GR can bind specific sequences called Glucocorticoid Response Elements (GREs). Binding to the GRE activates the recruitment of chromatin-remodeling complexes and coregulators. Negative GRE (nGRE) were also reported, which are responsible for trans-repression of the target genes through the binding of GR monomers. Binding to nGRE prevents the dimerization and allows for the recruitment of corepressors, thus preventing the transcription of target genes. GR can also mediate trans-repression of target genes by tethering via interactions with other transcription factors as NFκB or AP-1 without direct binding to DNA. GR: Glucocorticoid Receptor; GRE: Glucocorticoid Response Element; GILZ: Glucocorticoid-induced leucine zipper; SGK1: Serum and Glucocorticoid-Regulated kinase 1; PER 1: clock gene period 1; TM: Transcriptional Machinery.

Kidneys are important tissue targets of corticosteroid signaling pathways and play a crucial role in the neonatal period. Human neonates present with impaired sodium and water reabsorption during the first months of life, which is related to a partial tubular resistance to aldosterone [65] accompanied by high plasma aldosterone levels during the first months of life with progressive normalization to adult values [10]. Our group has shown that this transient and partial resistance to aldosterone in full-term healthy newborns is related to low tubular MR expression at birth, whereas MR is transiently expressed in the fetal kidney between the 14th and 24th GW [65]. Perinatal down-regulation of renal MR expression is not specific to the kidney, as it is also found in other mineralocorticoid target tissues such as the heart and the brain at variance with the lungs where MR expression is maintained at birth [100]. Interestingly, this temporal and tissue-specific expression of mineralocorticoid signaling is found both in mice and humans, demonstrating a well-conserved mechanism that may have a crucial role in the adaptation from aquatic in utero life to terrestrial life [65]. This variation in MR expression is not related to high aldosterone secretion at birth, since aldosterone synthase knockout mice present with the same neonatal down-regulation of renal MR expression [101]. However, all other players of the mineralocorticoid signaling pathway follow the same biphasic pattern of expression, such as the 11βHSD2 or the αENaC [65]. Interestingly, the down-regulation of 11βHSD2 in the kidney is not found in the placenta, where its expression is high during the prenatal period to protect the fetus against excessive impregnation by maternal glucocorticoids [66]. Although the mineralocorticoid signaling pathway is down-regulated during the perinatal period, the expression of GR is detected in renal tubular cells, and plasma cortisol levels are detectable in physiological amounts in newborns [66]. Given that 11βHSD2 is not detected, the renal glucocorticoid pathway is activated and cannot be regulated, thus supporting the idea of an equilibrium between mineralocorticoid and glucocorticoid signaling pathways during this specific period of development (Figure 2). To summarize, mineralocorticoid and glucocorticoid signaling pathways are tightly regulated during fetal life and exhibit cyclic periods of high and low activation, depending on the developmental stage. Mineralocorticoid signaling transiently decreases around the perinatal period whereas glucocorticoid secretion is low between 14 and 24 GW and increases exponentially prior to birth. Cyclic impregnation in mineralocorticoids and glucocorticoids seems to be part of the adaptation process to the extra uterine life.

## 8. Sexual Dimorphism in the Equilibrium between Renal Mineralocorticoid and Glucocorticoid Signaling

Several non-reproductive biological processes have a sexual dimorphic regulation. Blood pressure is one of the most well-recognized, with a differential of approximately 15 mmHg between systolic blood pressure in men and women before menopause [102]. This higher systolic blood pressure in males is conserved in all mammals, suggesting well-preserved regulatory mechanisms [103]. In adults, a direct effect of testosterone on blood pressure levels has been demonstrated in multiple animal models with castration and testosterone substitution experiments [104], whereas ovariectomy had no effect on blood pressure in female rats [105]. Sex steroids are known to influence the activity of the RAS in adults: testosterone promotes the action of Ang II via AT1R, whereas estrogen decreases the AT1R/AT2R ratio inducing a different receptivity to Ang II [103]. Our group observed a sex and organ-specific regulation of target genes of the corticosteroid signaling pathway in adult mice, along with a higher expression of renal 11βHSD2 in female mice, promoting the selectivity of aldosterone for its receptor [77]. This increased activation of the mineralocorticoid pathway in females does not increase blood pressure but could be aimed at a finer regulation of potassium excretion by distal tubules, which is an adaptive mechanism optimized for maternal–fetal homeostasis during pregnancy [106]. Of particular interest, Zheng et al., reported that the effects of aldosterone on plasma K^+^ were enhanced in females compared with males. These authors demonstrated that both Estrogen Receptors (ERα and ERβ) contributed to the estrogen-induced decrease in plasma K^+^ and AT1R binding in ovariectomized female rats [107]. Data in the developing fetus and newborn are less extensive. While no difference in *CYP11B1* and *CYP11B2* gene expressions or steroid concentrations of aldosterone and cortisol/corticosterone has been reported between male and female fetuses during development or at birth, sex specific MR and 11βHSD2 expression have been demonstrated [77]. Our group reported a sexual dimorphism in renal expression of the MR and its target genes during the perinatal period, with a peak in MR, GR, and mRNA expression of target genes at 17.5 days of gestation in female mice but not in males. These data are consistent with a previous study of Codon et al., showing a greater activity of the 11βHSD2 in female fetal kidney at 15 days of gestation [108]. It appears that in mice, the imbalance between MR and GR signaling pathways in the kidney during the prenatal period promotes mineralocorticoid signaling in females. This could confer an adaptive advantage for females, particularly in the lung, allowing the resorption of pulmonary fluid at birth by increased expression of αENaC [100]. Thus, the expression profile found in males could be interpreted as unfavorable and correlated with the greater morbidity presented by boys at birth, particularly in terms of respiratory adaptation [101]. Moreover, this suggests that the glucocorticoid signaling pathway may be preferentially activated in males, who may then be prone to pathological developmental programming following exposure to stress or glucocorticoids during gestation. 

## 9. Consequences in Pathophysiology

Given the imbalance between glucocorticoid and mineralocorticoid signaling pathways between males and females during the perinatal period, it is possible that this may have an impact under certain pathophysiological conditions, with a higher susceptibility for males to develop long term consequences. The “Developmental Origins of Health and Disease” hypothesis has caused resurgence of interest in understanding the factors regulating fetal development. A variety of prenatal perturbations may be involved in the onset of diseases in adulthood including cardiovascular and renal diseases. Our hypothesis is enforced by the existence of a gender difference in the incidence of cardiovascular diseases, such as high blood pressure and heart failure [109,110], which may be the consequence of early perinatal events [111].

### Fetal Growth Restriction

In humans, excess of maternal glucocorticoids causes a fetal growth restriction and a higher risk of hypertension later in life [112,113]. Studies (reviewed in [114]) using animal models (sheep, mouse, and rat models) of fetal growth restriction such as maternal glucocorticoid exposure, maternal calorie or protein restriction, and uteroplacental insufficiency, resulting either in a reduction in 11βHSD2 placental expression or direct fetal overexposure to glucocorticoids [115] (possibly inducing an overactivation of renal glucocorticoid signaling pathway) have identified alterations in kidney development as being a common feature. Interestingly, in many animal models of developmental programming, there is a sexual dimorphism between males and females in the timing of onset and severity of disease outcomes. Actually, the same prenatal insult does not always affect males and females similarly or to the same degree [114]. The formation of a low nephron endowment may result in impaired renal function and in turn may contribute to disease. These animal models develop programmed hypertension partially due to altered kidney development, resulting in a permanent reduction in offspring nephron endowment [116]. In humans, the number of nephrons is correlated with birth weight, with an estimated gain of approximately 237,426 nephrons per kilogram of additional birth weight, but it is more marked in males [117], which may lead to a differential developmental programming of hypertension between males and females. Importantly, the period of nephrogenesis varies among species with human and sheep completing nephron formation prior to birth, while rodents continue this developmental process after birth [116]. This means both the prenatal and postnatal environment can affect nephron endowment in the mouse. Aside from nephron endowment, modifications in the expression of different players of the corticosteroid signaling pathways have been demonstrated in these models [114], which are not always in association with a reduction in nephron number, thus suggesting that other mechanisms are implicated in developmental programming of high blood pressure [118]. AT1R and AT2R, which are expressed in the kidney early during gestation, have a sexually dimorphic altered expression in animal models of excessive glucocorticoid fetal impregnation, usually resulting in an increased expression of AT1R in males, depending on the timing of the prenatal insult [114]. Preliminary results from our group also suggest a decreased expression in renal MR expression under perinatal glucocorticoid overexposure, with the development of early hypertension, particularly in males.

- *Prematurity*

Preterm birth is associated with increased risks of mortality and morbidity [119]. Studies in preterm infants have demonstrated that males have higher risks of morbidities including respiratory distress syndrome, late-onset sepsis, bronchopulmonary dysplasia, and intraventricular hemorrhage, than females [120] and long term neurological consequences [121]. In addition, former preterm infants have a higher risk of developing early hypertension in adult life [122], particularly for preterm boys [123]. These sex differences are not linked to a variability in HPA axis function [33], but they may be in relation with a higher sensitivity to the administration of antenatal corticosteroids in boys [124]. In a model of lipopolysaccharide-induced prematurity, generated by our group, we observed that former preterm males develop significant hypertension in adulthood [125]. This hypertension is associated with early changes in the expression of different players of the corticosteroid signaling pathway during the neonatal period. Indeed, premature mice exhibited a very strong organ-specific renal activation of the expression of corticosteroid target genes (ENac, Sgk1, and Gilz), which contrasts with a significant decrease in renal MR expression. This suggests GR activation by glucocorticoids, which may program renal functional or molecular alterations, leading to hypertension in adulthood. The developmental programming of hypertension has been described by Barker et al. [126], and the mechanisms invoked were mainly nephron endowment, leading to compensatory hyperfiltration of existing nephrons with glomerulosclerosis and proteinuria in adulthood [127]. Few studies have demonstrated a sexual dimorphism in this prematurity-induced nephron reduction in humans, but no differences had been demonstrated in mice [128]. In our model, formerly premature male mice developed hypertension, independently from nephron number reduction in adulthood, suggesting other pathophysiological mechanisms involved. In addition, a study in humans suggested that the programming of hypertension could be transmitted to the children of former preterm infants; however, the small sample size did not allow distinguishing a sexual dimorphism [129]. In our mouse model, we identified a transmission of blood pressure dysregulation to subsequent generations from preterm neonates, up to the third generation. Interestingly, this vascular anomaly was only transmitted in males in the second and third generation, which is associated with a significant increase in expression of the corticosteroid target gene *Gilz* and a global hypomethylation of its promoter [125]. This study demonstrates that a predisposition to arterial hypertension could be epigenetically programmed in males by events occurring during the perinatal period in previous generations through sexually dimorphic adverse activation of corticosteroid signaling pathway.

- *Transient Pseudo-Hypoaldosteronism*

**Figure 3:**
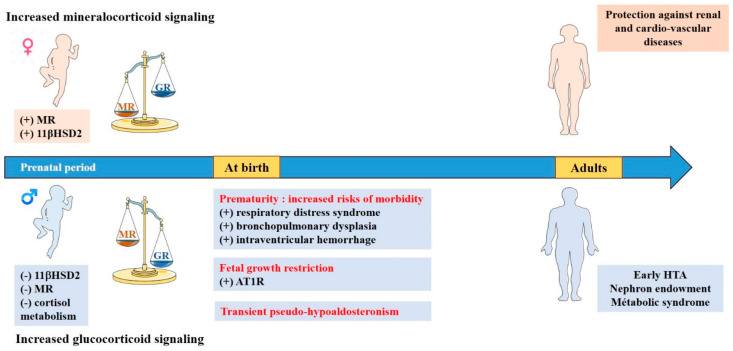
Sexual dimorphism of corticosteroid signaling during kidney development and consequences in pathophysiology. Sexual dimorphism of corticosteroid signaling is effective as early as the perinatal period with higher MR expression and 11βHSD2 activity in females and higher cortisol metabolism in males, thus favoring mineralocorticoid signaling in female fetus and neonates, and preferential activation of the glucocorticoid pathway in males. These imbalances may have an impact under certain pathophysiological conditions with a higher susceptibility for males to develop short and long term consequences after exacerbated activation of perinatal glucocorticoid signaling pathway.

During the early postnatal period, renal mineralocorticoid and glucocorticoid imbalance can also be challenged by urinary infection. Indeed, in case of upper urinary tract infection (pyelonephritis) with or without underlying uropathy, a transient, non-physiological pseudo-hypoaldosteronism may appear [130]. It results in hyponatremia, hyperkalemia, metabolic acidosis, and severe dehydration with major urinary sodium loss, requiring sodium supplementation in the acute phase. Transient pseudo-hypoaldosteronism has the particularity to occur mainly in infants under 3 months of age, in relation to the low renal MR expression at this period of development [65] and in 88% of the cases in males [130]. The pathophysiology may be related to inflammation (through the activation of NF-κB factor) that further downregulates MR expression and activation [131]. As MR expression is lower in males during the perinatal period [77], they appear more sensitive to a decline in its expression. In addition, the rise in glucocorticoid secretion induced by inflammation may trigger an overactivation of renal GR in males (that have lower 11βHSD2 levels) and induce alternative adverse effects. Overall, early perinatal events that will challenge renal corticosteroid signaling pathway may trigger short term and long term consequences in a gender-dependent manner. Figure 3 summarizes such renal corticosteroid imbalance between biological sexes and related disorders throughout development.

## 10. Conclusions

In summary, this review aimed at demonstrating the existence of a temporal window during renal development with a specific and temporal imbalance in glucocorticoid and mineralocorticoid signaling activation, along with a sexual dimorphic regulation. This sex-differential expression and activation of renal corticosteroid signaling pathways in the mammalian fetus and neonate, conserved among species, appear to favor mineralocorticoid signaling in females and glucocorticoid signaling in males. These differences may arise from direct or indirect effects of sex steroids; however, other mechanisms are likely to be at stake. Deciphering such regulatory mechanisms may shed light on short term and long term pathophysiological consequences, markedly for males, and contribute to improve the prevention and management of sex dimorphic diseases such as early hypertension.

## Figures and Tables

**Table 1:** MR and GR target genes in renal principal cells.

Target Genes	Nuclear Receptors	Functions	References
αENAC subunit	MR	Na^+^ transport	[57]
Na^+^, K^+^-ATPase	MR	Na^+^ transport	[58]
KS-WNK1	MR	Na^+^ transport	[60]
PER1	MR, GR	Circadian rhythm	[63]
NDRG2	MR	Cell differentiation	[61]
SGK1	GR, MR	Ser/Thr protein kinase ENAC trafficking/Na^+^ reabsorption	[59]
GILZ	GR, MR	ENAC trafficking/Na^+^ reabsorption	[62]
FKBP5	GR, MR	Chaperone protein	[67]

## Data Availability

Not applicable.
